# Detection of driver metabolites in the human liver metabolic network using structural controllability analysis

**DOI:** 10.1186/1752-0509-8-51

**Published:** 2014-05-03

**Authors:** Xueming Liu, Linqiang Pan

**Affiliations:** 1 Key Laboratory of Image Information Processing and Intelligent Control, School of Automation, Huazhong University of Science and Technology, Luoyu Road 1037, 430074 Wuhan, China

**Keywords:** Human liver metabolic network, Controllability, Driver metabolite

## Abstract

**Background:**

Abnormal states in human liver metabolism are major causes of human liver diseases ranging from hepatitis to hepatic tumor. The accumulation in relevant data makes it feasible to derive a large-scale human liver metabolic network (HLMN) and to discover important biological principles or drug-targets based on network analysis. Some studies have shown that interesting biological phenomenon and drug-targets could be discovered by applying structural controllability analysis (which is a newly prevailed concept in networks) to biological networks. The exploration on the connections between structural controllability theory and the HLMN could be used to uncover valuable information on the human liver metabolism from a fresh perspective.

**Results:**

We applied structural controllability analysis to the HLMN and detected driver metabolites. The driver metabolites tend to have strong ability to influence the states of other metabolites and weak susceptibility to be influenced by the states of others. In addition, the metabolites were classified into three classes: critical, high-frequency and low-frequency driver metabolites. Among the identified 36 critical driver metabolites, 27 metabolites were found to be essential; the high-frequency driver metabolites tend to participate in different metabolic pathways, which are important in regulating the whole metabolic systems. Moreover, we explored some other possible connections between the structural controllability theory and the HLMN, and find that transport reactions and the environment play important roles in the human liver metabolism.

**Conclusion:**

There are interesting connections between the structural controllability theory and the human liver metabolism: driver metabolites have essential biological functions; the crucial role of extracellular metabolites and transport reactions in controlling the HLMN highlights the importance of the environment in the health of human liver metabolism.

## Background

Metabolism is one of the most complex cellular processes and a basal system for maintaining life of all organisms. Liver metabolism disorders could cause a wide range of diseases, ranging from hepatitis to hepatic tumor [1]. Many studies which focus on the roles of single molecule substances or single paths in regulating liver metabolism have been carried out. For example, the interleukin receptor-associated kinase-M (IRAK-M) has been found to negatively regulate the innate and the adaptive immune response in the liver reacting to acute insult by alcohol [2]; the liver X receptors (LXRs) could bind to cholesterol metabolites and regulate the cholesterol turnover [3]; the metabolic changes in the glucose metabolism and the TCA cycle in liver have been found to be related to diabetes progression [4]. While an understanding of single molecules continues to be important, the focus is on understanding the whole metabolic network at a systems-level. Because a metabolic system is not just an assembly of metabolites, its properties cannot be fully understood merely by studying the single molecules [5].

With the accumulation of the relevant data, it becomes feasible to study metabolic systems in a genome-scale. A human metabolic model has been reconstructed based on genomic and bibliomic data [6]. The reconstructed genome-scale human metabolic model has been used to study human physiology and pathology [7]. Based on the human metabolic model [6] and a variety of different tissue-specific data, a human liver metabolic model has been derived [8]. For the method for the reconstruction and analysis of metabolic models, flux balance analysis (FBA) is a mathematical approach for analyzing the flow of metabolites through a metabolic network [9], which is widely used in predicting the rate of production of a bio-technologically important metabolite. When using FBA, the constructed models must satisfy the following requirements: models without gaps, electron balanced, mass balanced, etc. While for metabolic models created by some algorithms, such as INIT [10], they may not satisfy all the requirements. Even if the dissatisfaction exist, studies on these models could uncover novel valuable information on metabolic systems based on network analysis [11,12]. Thus, it is rewarding to study the metabolic systems from the perspective of networks.

Network science is an emerging field concerned with the study of complex systems represented as networks [13], which has become a powerful conceptual paradigm in the field of biology to understand biological systems at a systems-level [14,15]. In network science, how to control a system is a central issue [16]. Due to the unknown architecture of a system and the dynamical rules that capture the interactions between the components, it is difficult to control the complex system [16,17]. By fixing the weights of interactions between the components to be either 0 or free parameters, the structural controllability was defined and studied to show some connections between the control theory and network [18,19]. Liu et al. have used the theory of structural controllability to many models of real networks [16,17], and proved that by giving control signals to a minimum set of nodes (such nodes are called driver nodes), the whole network can be guided to any desired final state in finite time. Recently, structural controllability analysis has been applied to some biological networks, where interesting properties on the biological system and drug-targets have been discovered [12,20]. It is fair to expect that there are some possible connections between the structural controllability theory and the human liver metabolic network, which could provide valuable information on the human liver metabolism, such as the discovery of essential metabolites.

Abnormal states of the human liver metabolic network could lead to different metabolic diseases, such as diabetes [21], obesity [22] and cancers [23]. Sometimes, these abnormal states can be steered into normal states by different appropriate inputs: drugs, signals from environment or inside the organism, the injection of specific metabolites. Theses control inputs could lead to the changes in metabolic states (the concentration of metabolites) which influence the metabolic functions. For example, the drug raltitrexed can be used in cancer chemotherapy by targeting at the metabolite thymidylate synthase [24]; the injection of potassium can make the body functioning normal when the body suffers from the metabolic disorder of hypokalemia. If an organism suffers from metabolic disorders and the metabolic network cannot be controlled with any control inputs (drugs, signals from environment or inside the organism, etc.), then the organism may develop cancer or apoptosis. Researches on the controllability of the human liver metabolic network could provide the basis for ultimately understanding liver disease mechanisms, facilitating the development of therapeutics optimized for efficacy.

In this work, we applied the structural controllability analysis to the HLMN, detecting the metabolites and reactions that play important roles in the controllability of the HLMN. We identified driver metabolites in the HLMN, and classified the metabolites into three classes: critical, high-frequency and low-frequency driver metabolites. Among the 36 critical driver metabolites, 27 metabolites are essential, which suggests that the critical driver metabolites play important roles in the human liver metabolism. We find that the high-frequency driver metabolites tend to participate in different metabolic pathways, which are important in regulating the whole metabolic systems. The critical and high-frequency driver metabolites may be potential drug-targets. Moreover, we explored the other possible connections between the structural controllability theory and the HLMN. For example, by analyzing the roles of different links of the HLMN in the robustness of controllability, we find that transport reactions and the environment are important in the human liver metabolism. The results in this work show some connections between the structural controllability analysis and the human liver metabolism, which uncover valuable information on the human liver metabolism from a fresh perspective.

## Results and discussion

### Description of the human liver metabolic network

We used a human liver metabolic model represented by a set of metabolic reactions [8], which contains 1360 metabolites and 1826 reactions. The human metabolic model was generated based on MBA algorithm [8], which is a model-building algorithm used to derive tissue-specific metabolic models from a generic model [6] by integrating a variety of tissue-specific molecular data sources, including literature-based knowledge, transcriptomic, proteomic, metabolomic and phenotypic data. In the human liver metabolic model, each metabolite is represented in the form of *A*[*x*], where *A* is the name of a metabolite and *x* in the bracket [ ] is the abbreviation of the cell compartment where the metabolite *A* appears (see Additional file 1). Metabolite *A* may appear in different cell compartments *x*,…,*y*, so there are *A*[*x*],…,*A*[*y*] for the same metabolite but different cell compartments, which are counted as different metabolites.

**Figure 1:**
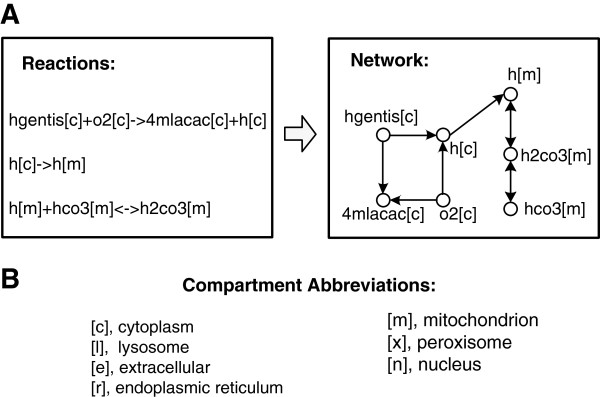
**An example to show how the HLMN is reformatted from the liver model.** **A)** Three metabolic reactions in the liver model are shown on the left, where hgentis, o2, 4mlacac, h, hco3 and h2co3 are metabolites, [c] and [m] are the abbreviations of cell compartments "cytoplasm" and "mitochondrion" denoting where the corresponding metabolites appear. The first metabolic reaction represents that homogentisate in cytoplasm (hgentis[c]) is oxidated into 4-Maleylacetoacetate (4mlacac[c]) and hydrogen ion (h[c]); the second means that the hydrogen ion in cytoplasm (h[c]) is transported into mitochondrion (h[m]); the third represents that the hydrogen ion in mitochondrion (h[m]) reacts with bicarbonate (hco3[m]) to form carbonic acid (h2co3[m]). The network reformatted from these three metabolic reactions is shown on the right, where each node denotes a metabolite with the information of cell compartment where it appears, two nodes have a link if there is a chemical reaction such that one metabolite is substrate and another one is a product. **B)** The abbreviations of cell compartment and their corresponding full names.

Based on the principle that a set of metabolic reactions can be translated into a network representation [25], we reformulated the liver model in the following way: denoting each metabolite by a node labeled with *A*[*x*], and connecting two nodes by *A*[*x*] → *B*[*y*] if there is a chemical reaction where *A*[*x*] is a substrate and *B*[*y*] is a product. The derived HLMN contains 1360 nodes and 6501 links (see Additional file 1). In order to illustrate the process of reformulating the HLMN, an example with three metabolic reactions is given in Figure 1.

For convenience and without ambiguity, we will not distinguish nodes from metabolites hereinafter when refer to the properties of the HLMN. For example, when we say a driver node in the HLMN, we may mean a driver metabolite in the HLMN.

### Classification and analysis of driver metabolites

Driver metabolites in the HLMN are metabolites where inputs are injected. If the driver metabolites in a minimum driver metabolites set (MDMS, for short) are all controlled by different inputs, the HLMN can be steered from any given state to a desired state in finite time. "Minimum" means that if signals are only input on a proper subset of *S*, then the HLMN cannot be guided to some final desired states in finite time. MDMSs are determined by detecting maximum matchings in the HLMN (see Methods).

A maximum matching is a maximum set of links that do not share start or end nodes [16]. There are different maximum matchings in a network [26], which could result in different MDMSs in the HLMN. Counting the number of all maximum matchings in an arbitrary network has been proven to belong to the ♯P-complete (sharp P-complete) class of problems [27]. There is no currently known polynomial-time algorithm for solving a ♯P-complete problem. The number of maximum matchings can grow exponentially with networks size, hence a network with only hundreds of nodes often leads to millions of maximum matchings. Enumeration of maximum matchings is computationally prohibitive for large networks [28]. Thus, the enumeration of maximum matchings in the HLMN (containing 1360 nodes) is hard to achieve.

#### Classification of driver metabolites

We randomly identified 5000 different maximum matchings (see Additional file 2) and their corresponding MDMSs (see Methods). In the HLMN, a node may appear in different MDMSs. For each node *v*, we counted the number of MDMSs that the node *v* appears in and then normalized the number (that is, the number is divided by 5000). The normalized values characterize the frequency *f*_*d*_ of each node appearing in the 5000 MDMSs. According to the frequency of each node, we classified the metabolites into three groups: critical driver metabolites with *f*_*d*_ = 1, high-frequency driver metabolites with 0.6 ≤ *f*_*d*_ < 1, low-frequency driver metabolites with 0 ≤ *f*_*d*_ < 0.6.

A node with *f*_*d*_ = 1 means that the node appears in all the MDMSs. Such nodes may possess some specific properties or functions, which could provide valuable information on the HLMN. So we classified the nodes with *f*_*d*_ = 1 being critical driver nodes. The reason why we chose the threshold 0.6 to separate high-frequency driver metabolites from low-frequency driver metabolites, is that we would like to make the difference between the roles of metabolites in these two groups as big as possible (for detailed analysis, see the subsection "The roles of the high-frequency driver metabolites").

In order to test whether the classification of metabolites based on 5000 MDMSs is reliable, we computed the frequencies of metabolites in 51 different families of MDMSs with sizes of 5000,5100,5200,…,10000. The frequency of each metabolite computed based on different families of MDMSs stays in a same region, where the regions are *f*_*d*_ = 1, 0.6 ≤ *f*_*d*_ < 1 and 0 ≤ *f*_*d*_ < 0.6 (see Additional file 3). In other words, the classifications of each metabolite are the same based on these different families. Hence the classification of metabolites based on 5000 MDMSs is reliable. Furthermore, we have employed an unbiased random sampling method [28] to validate the results based on the 5000 MDMSs (for detailed analysis, see the subsection "Validation for the classification and the properties of driver metabolites").

#### Topological analysis of driver metabolites in the HLMN

We computed different centralities of each metabolite *i* in the HLMN, which include out-degree *OutD*, in-degree *InD*, degree *D*, betweenness *BC*, closeness *CC*, in-closeness *CCI* and out-closeness *CCO* (for definitions, see Methods). The frequency *f*_*d*_ was found to decrease quickly with the in-degree (see Additional file 4) while this pattern does not hold for other centralities, which is consistent with the result in [28]. For each centrality, all metabolites in the HLMN are divided into three sets of similar sizes, based on their centrality scores (low, medium, and high), In this way, seven families of sets were obtained: $F_{D}=\{D_{l},D_{m},D_{h}\}$, $F_{\text{OutD}}=\{{\text{OutD}}_{l},{\text{OutD}}_{m},{\text{OutD}}_{h}\}$, $F_{\text{InD}}=\{{\text{InD}}_{l},{\text{InD}}_{m},{\text{InD}}_{h}\}$, $F_{\text{BC}}=\{{\text{BC}}_{l},{\text{BC}}_{m},{\text{BC}}_{h}\}$, $F_{\text{CC}}=\{{\text{CC}}_{l},{\text{CC}}_{m},{\text{CC}}_{h}\}$, $F_{\text{CCI}}=\{{\text{CCI}}_{l},{\text{CCI}}_{m},{\text{CCI}}_{h}\}$, $F_{\text{CCO}}=\{{\text{CCO}}_{l},{\text{CCO}}_{m},{\text{CCO}}_{h}\}$, where each family contains three sets, and the subscripts *l*,*m*,*h* respectively represent low, medium and high.

**Figure 2:**
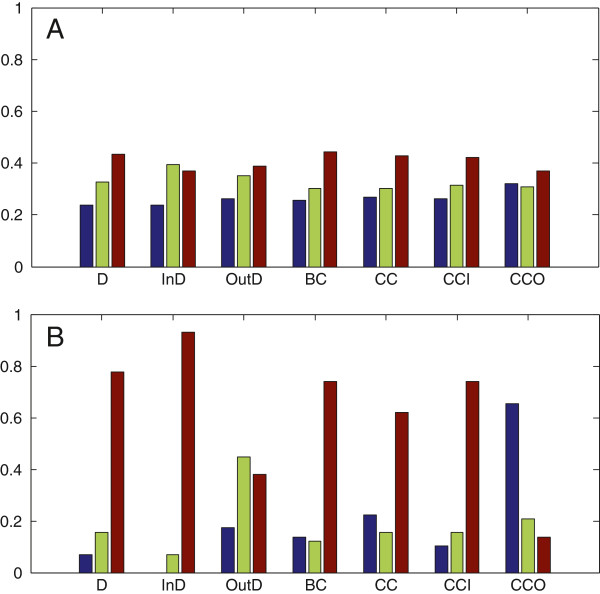
**Topological analysis of the driver metabolites.** **A)** The metabolites in set A (the union of metabolites from the 5000 MDMSs); **B)** The metabolites in set B (the set of critical and the high-frequency driver metabolites). For the labels in the horizontal axis, "D", "InD", "OutD", "BC", "CC", "CCI", "CCO" respectively represent degree, in-degree, out-degree, betweenness, closeness, in-closeness, out-closeness. The hight of blue, green and brown bars respectively represent the fractions of the driver metabolites with high, medium and low centrality scores. The difference between the fractions for each centrality in B) is greater than that in A). Except for the out-closeness in B), the brown bars are all lower than the blue and green ones for the same centrality, which indicates that the driver metabolites tend to avoid nodes with the high degree (resp., out-degree, in-degrees, betweenness, closeness and in-closeness), while the critical and high-frequency driver metabolites tend to have high out-closeness.

We used set *A* to denote the union of metabolites from the 5000 MDMSs, and set *B* to denote the union set of the critical and high-frequency driver metabolites. For each of the families $F_{D},F_{\text{OutD}},F_{\text{InD}},F_{\text{BC}},F_{\text{CC}},F_{\text{CCI}},F_{\text{CCO}}$, the fractions of metabolites from set *A* that belong to the three sets in the family were computed (see Figure 2(A)), and the fractions of metabolites from set *B* that belong to the three sets in the family were also computed (see Figure 2(B)). For example, for the family $F_{D}$, we computed |*A* ∩ *D*_*l*_|/|*A*|, |*A* ∩ *D*_*m*_|/|*A*|, |*A* ∩ *D*_*h*_|/|*A*|, and |*B* ∩ *D*_*l*_|/|*B*|, |*B* ∩ *D*_*m*_|/|*B*|, |*B* ∩ *D*_*h*_|/|*B*|, where |∗| denotes the size of set ∗.

Comparing the results shown in Figure 2(A) and Figure 2(B), we find that for each centrality, the difference between the fractions computed in set *B* is greater than that in set *A*, which means that the topological characteristic differences are bigger in the set of critical and high-frequency driver metabolites. In-degree and in-closeness measure the susceptibility of a metabolite to be influenced by other metabolites. Higher in-degree and higher in-closeness imply that the metabolite could be more easily influenced by others. Out-closeness measures the ability of a metabolite to influence other metabolites. Higher out-closeness implies that the metabolite could influence others more easily. The metabolites in set *B* tend to have low in-degree, low in-closeness, and high out-closeness. Therefore, the driver metabolites, especially the critical and high-frequency driver metabolites, tend to have strong ability to influence the states of other metabolites and weak susceptibility to be influenced by the states of other metabolites. Moreover, injecting control inputs (drugs, signals from environment or inside the organism, etc.) to critical and high-frequency driver metabolites could regulate the whole state of the HLMN, which indicates that the critical and high-frequency driver metabolites may be potential drug-targets.

For each centrality, we used chi-square test (see Methods) to establish whether or not the fraction distribution in set A and set B differs from that in the whole network (the reason why we chose chi-square test is given in Methods). The chi-square statistic values for each centrality in set A and set B are shown in Table 1. While the table value for chi-square statistic is 5.99, based on the freedom being 2 and the level of significance being 0.05. Except for the *CCO*, other chi-square statistic values are greater than the table value in set A, and the chi-square statistic values for all the centralities are greater than the table value in set B. It means that except for the *CCO* in set A, for other centralities in set A and all the centralities in set B, the fraction distributions differ from that in the whole network. Thus, the result of the topological features of driver metabolites is of statistical significance.

**Table 1:** The chi-square statistic value for different centralities in set A and set B

	***D***	***InD***	***OutD***	***BC***	***CC***	***CCI***	***CCO***
Set A	32.37	24.14	14.31	33.21	25.03	22.07	3.68
Set B	51.86	93.80	7.13	43.58	22.03	43.78	27.51

#### Properties of the critical driver metabolites

In the HLMN, we detected 36 critical driver metabolites (see Table 2). Their in-degrees are all zero, which is consistent with the result in [29] and means that the 36 critical driver metabolites are all the start metabolites of paths (paths in the HLMN are sequential reactions between metabolites). By Lin’s structural controllability theorem [18,30], if a system is controllable, there is no inaccessible nodes (i.e., nodes that cannot be accessed or "influenced" by the external inputs). Since these start metabolites cannot be influenced by the external inputs via other metabolites, they need to be directly controlled by external inputs.

**Table 2:** The list of the critical driver metabolites in the HLMN

**Metabolite name**	**Metabolite description**	**Category**	**In-degree**
5dhf[e]	pentaglutamyl folate (DHF)	FEM [31]	0
5thf[e]	pentaglutamyl folate (THF)	FEM [31]	0
ach[e]	Acetylcholine	FEM [32]	0
adp[e]	ADP	UM [33]	0
arg-DASH-L[e]	L-Arginine	UM [33]	0
asp-DASH-L[e]	L-Aspartate	UM [33]	0
atp[e]	ATP	UM [33]	0
avite1[e]	alpha-Tocopherol	FEM [34]	0
biocyt[e]	Biocytin	FEM [35]	0
cmp[e]	CMP	UM [33]	0
fe3[e]	Fe 3+	FEM [36]	0
glygn2[e]	glycogen, structure 2	EUM	0
gtp[e]	GTP	UM [33]	0
ha[e]	hyaluronan	FEM [37]	0
idp[e]	IDP	FEM [38]	0
ksi[e]	keratan sulfate I	EUM	0
lcts[e]	Lactose	EUM	0
Lcystin[e]	L-Cystine	FEM [39]	0
met-DASH-L[e]	L-Methionine	UM [33]	0
nac[e]	Nicotinate	EUM	0
nad[e]	Nicotinamide adenine dinucleotide	UM [33]	0
nadp[e]	Nicotinamide adenine dinucleotide phosphate	UM [33]	0
orn[e]	Ornithine	EUM	0
paf-hs[e]	1-alkyl 2-acteylglycerol 3-phosphocholine	FEM [40,41]	0
pe-hs[e]	phosphatidylethanolamine	FEM [42,43]	0
pglyc-hs[e]	phosphatidylglycerol	EUM	0
pnto-DASH-R[e]	(R)-Pantothenate	FEM [44]	0
ppa[e]	Propionate (n-C3:0)	FEM [45]	0
s2l2fn2m2masn[e]	PA6	EUM	0
strch1[e]	starch, structure 1	FEM [46]	0
sucr[e]	Sucrose	FEM [47]	0
tagat-DASH-D[e]	D-Tagatose	EUM	0
tag-hs[e]	triacylglycerol	FEM [48]	0
ttdca[e]	tetradecanoate (n-C14:0)	EUM	0
utp[e]	UTP	UM [33]	0
yvite[e]	gamma-Tocopherol	FEM [49,50]	0

The compartment abbreviation of all critical driver metabolites are "[e]", which means that their locations are all extracellular. The in-degree of each critical driver metabolite is zero, which implies that each critical driver metabolite is the start metabolite of a metabolic pathway. The categories "UM", "FEM", "EUM" respectively represent the universal metabolites, the functional essential metabolites and the metabolites whose essentiality have not been discovered.

The 36 critical driver metabolites are all found to be extracellular (each of the 36 critical driver metabolites is associated with the abbreviation of compartmental information "[e]", which means extracellular). By checking the biochemistry activities of the 36 critical driver metabolites, we find that they all participate in the transport reactions from the extracellular into the cell, which suggests that the intakes of these extracellular metabolites play important roles in the biological activities of the liver cells. For example, appropriately increasing the intake of the critical driver metabolite gamma-tocopherol could help lower the cholesterol level, and increasing the intake of the critical driver metabolite alpha-tocopherol could decrease lipid peroxidation and hepatic stellate cells activation, which could protect liver cells and prevent liver fibrosis [51].

We investigated the biological essentiality of the 36 critical driver metabolites. The essentiality of a metabolite measures how important the metabolite is in the whole metabolic systems or some metabolic processes. Although a metabolite could exist in different compartments, the metabolite is recognized to be essential as long as it is found to be essential in any one of the compartments [33]. Based on the different essentiality of metabolites, the metabolites were classified into three groups: 

• Universal Metabolites (UM): Some inorganic or cofactor metabolites, such as CMP and ATP, which have been found to exist universally in more than 90% organisms. The universal metabolites are usually treated as essential metabolites because most living matter cannot survive without them [33,52].

• Functional Essential Metabolites (FEM): The metabolites which are not UM and have essential roles in some biological functions. For example, folate is essential to numerous bodily functions, and required by the human body to synthesize, repair and methylate DNA as well as to act as a cofactor in certain biological reactions [31]; Hyaluronan is essential for embryogenesis [37]; Human body requires pantothenic acid to synthesize coenzyme-A (CoA), as well as to synthesize and metabolize proteins, carbohydrates, and fats [44].

• Essentiality Undiscovered Metabolites (EUM): The metabolites whose essentiality have not been discovered. These metabolites may be the potential essential metabolites, which demands further experimental verification.

Among the 36 critical driver metabolites, we find that 10 metabolites are UM; 17 metabolites are FEM; 9 metabolites are EUM. Therefore, among the 36 critical driver metabolites, 27 metabolites are essential, which suggests that the critical driver metabolites play important roles in human liver metabolism.

#### The roles of the high-frequency driver metabolites

We used simulated annealing (SA) algorithm [53] to detect modules in the HLMN. The reason why we chose the SA algorithm is that it is a commonly used technique to detect modules, and a benchmark to validate the effectiveness of the newly developed module-detecting algorithms [54,55]. Compared with other module-detecting algorithms, such as the markov clustering method, the SA algorithm performs better in detecting modules in large scale metabolic networks and the detected modules are more biologically meaningful [56], since the SA algorithm is less sensitive to noise such as experimental error or incomplete data.

According to the two parameters within-degree and the partition coefficient of each node in the modularized HLMN, the nodes were divided into seven classes: R1, R2, R3, R4, R5, R6, R7 (for details, see Methods).

**Figure 3:**
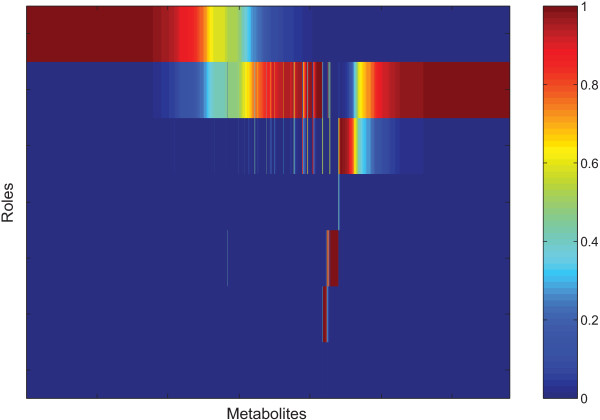
**The probability of each metabolite for each role among 100 partitions.** The color is mainly dark red (the corresponding probability equals to 1) or dark blue (the corresponding probability equals to 0). It means that most metabolites in the HLMN are always classified into a same role class among 100 partitions, which indicates that the role classification for metabolites is reliable.

Since the SA algorithm is stochastic, different results of modularization could be obtained in different runs. We have run the SA algorithm for 100 times. Based on the result of each run, the nodes of the HLMN were classified into the seven classes R1, R2, R3, R4, R5, R6, R7. Among the 100 classification results, the probability of each node being classified into each class is counted. As shown in Figure 3, most nodes are always classified into a same class, which indicates that the role classification for the nodes in the HLMN based on the SA algorithm is reliable.

**Figure 4:**
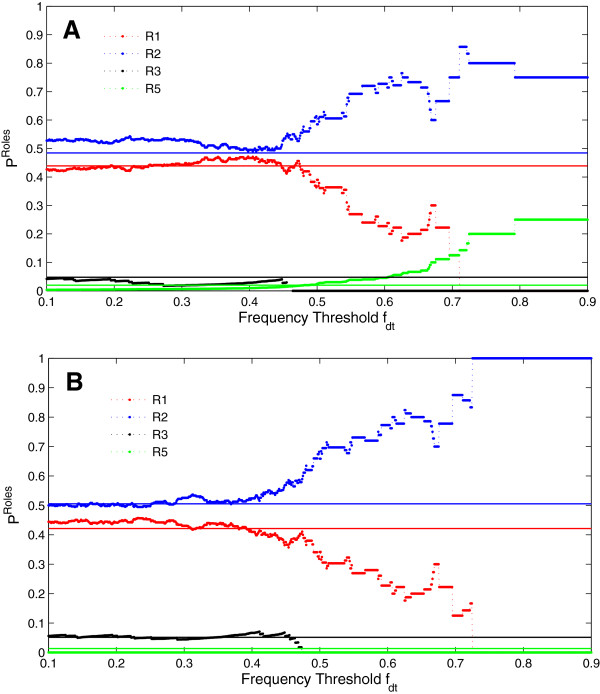
**The fractions of the metabolites with different roles based on different frequency threshold.** **A)** and **B)** respectively show the results which are based on the modules detected by the simulated annealing algorithm and the fast greedy algorithm. Each point connected by dotted lines is the fraction of the metabolites with a specific role among the set of driver metabolites whose frequency *f*_*dt*_ ≤ *f*_*d*_ < 1, while each solid line means the fraction of metabolites with each role among the HLMN. In the HLMN, most metabolites are of roles R1 and R2. With the frequency threshold *f*_*dt*_ increasing, the fraction of R1 metabolites among the set of metabolites with *f*_*dt*_ ≤ *f*_*d*_ < 1 decreases while the fraction of R2 metabolites increases. The fractions of metabolites with different roles fluctuate when *f*_*dt*_ ≥ 0.7 due to the small size of the set of metabolites with *f*_*dt*_ ≤ *f*_*d*_ < 1. When *f*_*dt*_ < 0.7, the difference between the fractions of R1 metabolites and R2 metabolites is the biggest at about *f*_*dt*_ = 0.6. Thus, we choose the threshold *f*_*dt*_ = 0.6 to differentiate the high-frequency driver metabolites from the low-frequency driver metabolites, and the high-frequency driver metabolites tend to be of role R2.

It has been found that the non-hubs connecting different modules are responsible for inter-module fluxes which influence the state of metabolic networks [57], while the nodes with high frequency *f*_*d*_ have strong ability to influence the states of other metabolites, which prompts us to think whether the nodes with high frequency *f*_*d*_ tend to be non-hubs connecting different modules. In the HLMN, more than 92% nodes are of roles R1 and R2, which are both non-hubs and R1 nodes have no connection with other modules while R2 nodes have connections with different modules. As shown in Figure 4(A), with the frequency threshold *f*_*dt*_ increasing, the fraction of R1 nodes among the set of nodes with *f*_*dt*_ ≤ *f*_*d*_ < 1 decreases while the fraction of R2 nodes increases. The fractions of nodes with different roles fluctuate when *f*_*dt*_ ≥ 0.7 due to the small size of the set of nodes with *f*_*dt*_ ≤ *f*_*d*_ < 1. When *f*_*dt*_ < 0.7, the difference between the fractions of R1 nodes and R2 nodes is the biggest at around *f*_*dt*_ = 0.6. Therefore, we chose the threshold *f*_*dt*_ = 0.6 to differentiate the high-frequency driver metabolites from the low-frequency driver metabolites. The fact that the roles of high-frequency driver metabolites tend to be R2, indicates that the high-frequency driver metabolites tend to be non-hubs connecting different modules. Different modules could be mapped to different pathways [56], which means that the high-frequency driver metabolites tend to participate in different metabolic pathways. For example, the high-frequency driver metabolite cyclic adenosine monophosphate plays regulatory roles in glucose, protein and fatty metabolism pathways at the same time [58]. It suggests that the high-frequency driver metabolites play important roles in human liver metabolic network.

To validate that the result of the high-frequency driver metabolites does not depend on the module detecting method SA algorithm, we used another module detecting method fast greedy [59] to detect modules in the HLMN, and classify the nodes into 7 classes: R1, R2, R3, R4, R5, R6, R7. With the frequency threshold *f*_*dt*_ increasing, the fractions of R1 nodes and R2 nodes among the set of nodes with *f*_*dt*_ ≤ *f*_*d*_ < 1 show the similar pattern as that based on the SA algorithm, which is shown in Figure 4(B). We arrived at the same conclusion that the high-frequency driver metabolites tend to be the non-hub connecting different modules.

#### Validation for the classification and the properties of driver metabolites

The results of the properties on the driver metabolites, critical driver metabolites and high-frequency driver metabolites are all based on the 5000 MDMSs. To validate that these results do not depend on the 5000 MDMSs, we applied an unbiased sampling method proposed by Jia et al. [28] to compute the frequency *f*_*d*_ that each node acts as a driver node (see Additional file 5).

Comparing with the results which based on the 5000 MDMSs, the set of critical driver nodes determined by this method is the same, while the set of high-frequency driver nodes determined by this method is not exactly the same, which may be caused by the randomness of sampling. However, the following result holds for both two methods: the high-frequency driver nodes tend to be the non-hubs connecting different modules. (see Additional file 4). Moreover, the topological analysis has been applied to the set A (the set of the metabolites with *f*_*d*_ > 0) and set B (the set of the metabolites with 1 ≤ *f*_*d*_ > 0.6) detected by the method in [28]. The conclusion still holds that the driver metabolites, especially the critical and high-frequency driver metabolites, tend to have strong ability to influence the states of other metabolites and weak susceptibility to be influenced by the states of other metabolites (see Additional file 4).

In conclusion, although the classification and analysis of driver metabolites are based on the 5000 MDMSs, the results on the properties of different driver metabolites do not rely on the 5000 MDMSs.

### Alternative classification of driver nodes and the control mode of the HLMN

A recently published paper [29] has given an alternative classification of nodes based on their participation in control. A node is critical, itermittent or redundant if it acts as a driver node in all, some or none of the minimum sets of driver nodes. By measuring the fraction *n*_*r*_ of the redundant nodes for a network with varying average degree, two distinct control modes were discovered in [29]. Based on the difference value of the fraction *n*_*r*_ and $n_{r}^{T}$ for its transpose network (whose wiring diagram is identical to the original network but the direction of each link is reversed), the control mode of a network can be identified: if $\Delta n_{r}=n_{r}-n_{r}^{T}>0$ the network is centralized and if Δ*n*_*r*_ < 0 it is distributed.

We have applied the tools in [29] to the HLMN, and find that the control mode of the HLMN is distributed. While in [29], the control modes of the three involved metabolic networks cannot be identified, which is caused by the incompleteness of the metabolic networks, whose average degrees are in the ‘pre-bifurcation’ region (where no distinct control modes exist). With more information on these metabolic networks being uncovered, the average degrees increase and result in identifiable control modes. For example, the E. coli metabolic network [11] studied in [29] was assembled in 2000, and its control mode cannot be identified; however, when we applied the tools to the E. coli metabolic network iJO1366 [60], which was assemble in 2011, we can find that the control mode of network iJO1366 is centralized. It is not easy to figure out the reason why the control mode of the human liver metabolic network is distributed and the E. coli metabolic network iJO1366 is centralized, due to the incompleteness of these two networks, whose control mode may alter with the increase of the network scale.

### The role of reactions in the robustness of the controllability in the HLMN

Reaction failures could happen in metabolic systems, and different reaction failures have different impacts on the robustness of the metabolic function. Robustness characters the ability of metabolic systems to behavior normally under reaction failures. Some reaction failures would break the cellular homeostasis, resulting in an anti-proliferative effect [61] or apoptosis [62], while some almostly have no influence on the cellular functions [63]. In what follows, we focus on the impacts of different reaction failures on the robustness (whether the network is controllable with the same MDMS under reaction failures) of the controllability in the HLMN.

**Figure 5:**
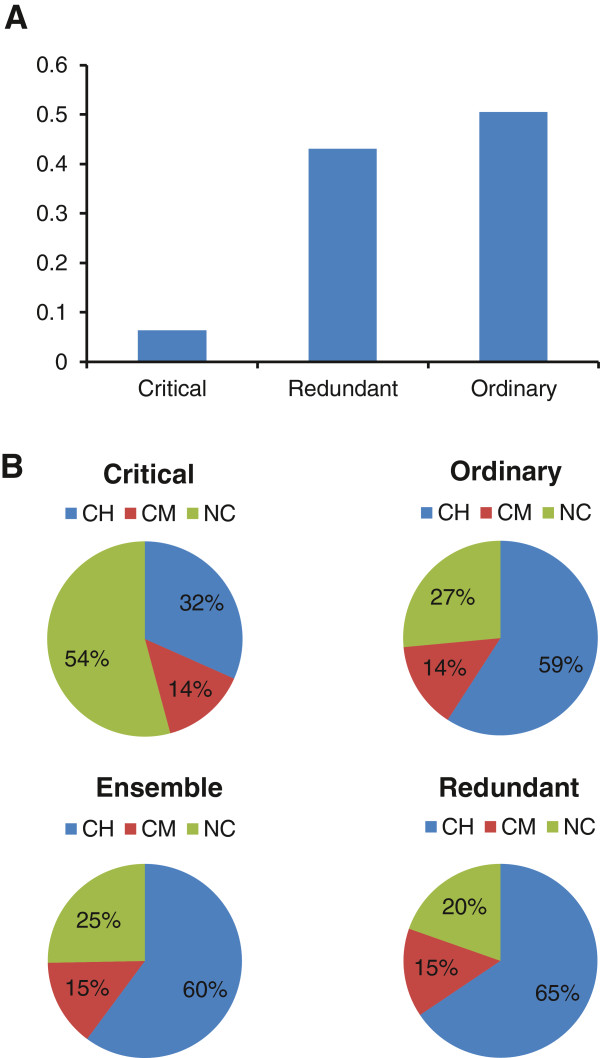
**The fractions of the links of different types.** The fractions of critical, ordinary and redundant links among the whole link set in the HLMN are shown in **A)**. Few links are critical and most links are ordinary, which implies that there are only a few reactions whose failure could cause the increase of the minimum number of driver nodes. The fractions of the core high (CH), core moderate (CM) and non-core (NC) reaction links among the set of critical, ordinary, redundant links or the set of all the links (Ensemble) are shown in **B)**. The fraction of the core high reaction links is the smallest and the fraction of non-core reaction links is the biggest in the set of critical links, which means that the reactions represented by critical links tend to be the non-core reactions in the human liver metabolic network.

Based on different impacts on the robustness of controllability caused by links absence, the links have been classified into three categories [16]: "critical" if its absence causes the minimum number of driver nodes increased so as to maintain full control; "redundant" if it can be removed without affecting the current set of driver nodes; "ordinary" if it is neither critical nor redundant. From the fractions of critical, ordinary and redundant links in the HLMN, which are shown in Figure 5, we can find that few links are critical and most links are ordinary, whose absence may change the current set of driver nodes, but the network could still be controlled with the same number of driver nodes. In the human liver metabolism, there are only a few reactions represented by critical links, which provides an explanation to why human liver metabolism could function well under many different circumstances.

In the human liver metabolic model [8], the reactions have been classified into three classes: core high reactions for these reactions included in human-curated tissue-specific pathways, which are essential in the human liver metabolism; core moderate reactions for these reactions testified by molecule data; non-core reactions for the other, most of which are not associated with genes in the model and 50% are transport reactions. We computed the fractions of links representing the core high, core moderate and non-core reactions among the set of critical, ordinary and redundant links and the set of all links in the HLMN, which are shown in Figure 5. Comparing with the fractions among the sets of the ordinary, redundant links and the whole link set in the HLMN, the fraction of links representing core high reactions are the lowest and the fraction of non-core reaction links are the highest in the set of critical links, which indicates that the reactions represented by critical links tend to be the non-core reactions.

Transport reactions transfer metabolites across compartments, many of them transfer metabolites from the environment into the cell. The fraction of transport reaction links among the set of critical links is 47.5%, while that among the whole link set in the HLMN is 20.8%. Moreover, we computed the fraction of links representing transport reactions which transfer metabolites from the environment into the cell among the set of critical links and that among the whole link set in the HLMN, which are 33.6% and 12.2%, respectively. These comparisons indicate that transport reactions and the environment are important in influencing the robustness of controllability of the HLMN. The metabolites carried in by transport reactions could activate a series metabolic reactions in human liver cells, which could change the state of the liver metabolism and influence the controllability of the HLMN.

#### Validation for the result that the reactions represented by critical links tend to be the non-core reactions

We used chi-square test (see Methods) to test whether or not the differences between the fractions of the core high, core moderate and non-core reaction links among the whole network and those among the set of critical links are out of chance. The observed data are the number of core high, core moderate and non-core reaction links among each of the sets of the critical links, which are 132, 59 and 226 respectively. The expected percentages are the fractions of core-high, core moderate and non-core reaction links among the whole network, which are 60.14% and 14.55% and 25.3% respectively. The chi-square statistic value was computed based on the chi-square formula (see Methods), which is 193.9. With the freedom degree being 2 and the significance level being 0.05, the table value for chi-square statistic is 5.99. The chi-square statistic value is bigger than the table value, so there is a significant difference between the fractions among the set of critical links and those among the whole network, which means that the reactions represented by critical links tend to be the non-core reactions.

## Conclusions

In this study, we have detected the driver metabolites in the HLMN and classified the metabolites into three classes: critical, high-frequency and low-frequency driver metabolites. Among the 36 critical driver metabolites, 27 metabolites are essential, which suggests that the critical driver metabolites play important roles in the human liver metabolism. Moreover, the compartments where the critical driver metabolites appear are all extracellular. It is consistent with our knowledge that the substances imported from the environment play important roles in steering the behavior of the whole metabolic network. The liver metabolic system could be regulated by controlling the intakes of the critical driver metabolites. For example, the increase of the intake of the critical driver metabolite alpha-tocopherol could decrease lipid peroxidation and hepatic stellate cells activation, so as to protect liver cells and prevent liver fibrosis [51]. We find that the high-frequency driver metabolites tend to participate in different metabolic pathways, which are important in regulating the whole metabolic systems. For example, the high-frequency driver metabolite cyclic adenosine monophosphate, which acts as a second messenger in many biological processes, plays important regulatory roles in glucose, protein and fatty metabolism pathways at the same time [58]. In addition, the states of the critical and high-frequency driver metabolites have strong ability in steering the state of the whole HLMN, indicating that the critical and high-frequency driver metabolites may be potential drug-targets.

By analyzing the roles of different links in the robustness of controllability, we find that transport reactions and the environment are important in the robustness of controllability in the HLMN under reactions failures. The metabolites carried in by transport reactions could activate a series metabolic reactions in human liver cells, leading to changes in the state of liver metabolism.

Moreover, we have explored some other possible connections between the structural controllability theory and the HLMN. Based on the structural controllability theory, two key concepts control centrality [64] and control mode [29] have been proposed. The control centrality of a node measures the number of nodes that can be independently controlled by controlling this node alone. We attempted to reveal the possible connections between the control centrality and the actual importance of a metabolite in the HLMN, but we find there is no such connection (see Additional file 4 and 6). We also applied the tools in [29] to give an alternative node classification in the HLMN based on node’s participation in control, and find that the control mode of the HLMN is distributed. It is not easy to figure out the reason why the HLMN is distributed, due to the incompleteness of this network, whose control mode may alter with the increase of the network scale.

In summary, we find that the driver metabolites have essential biological functions, and the metabolites connecting different pathways play crucial roles in the controllability of the HLMN. The crucial role of extracellular metabolites and the transport reactions highlight the importance of the environment in the health of human liver metabolism. The work presented here raises a number of questions. For example, what properties do the low-frequency driver metabolites have? How can we quantify the influence of each driver metabolite on the state of HLMN? Answers to these questions could further provide theoretical foundation for designing experiments of regulating the human liver metabolism.

## Methods

### Identification of driver metabolites

**Figure 6:**
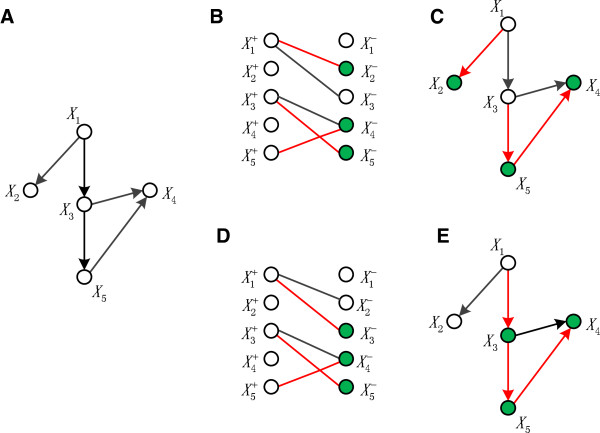
**The detection of driver nodes in a directed network.** The simple directed network in **A)** can be converted to the bipartite network in **B)** and **D)**. The links in red in B) and D) are two different maximum matching in the bipartite network, and the green nodes are the matched nodes. Mapping the bipartite network B) and D) back into the directed network, two different minimum sets of driver nodes are obtained, i.e., the sets of white nodes respectively shown in **C)** and **E)**.

Driver metabolites are detected by finding the maximum matchings in the HLMN. Matching is a set of links, where the links do not share start or end nodes. A maximum matching is a matching with maximum size. A node is matched if there is a link in maximum matching pointing at it; otherwise, it is unmatched [16]. A network can be fully controlled if every unmatched node gets directly controlled and there are directed paths from input signals to all matched nodes [65]. An example to find maximum matchings and detect MDMSs is shown in Figure 6.

**Figure 7:**
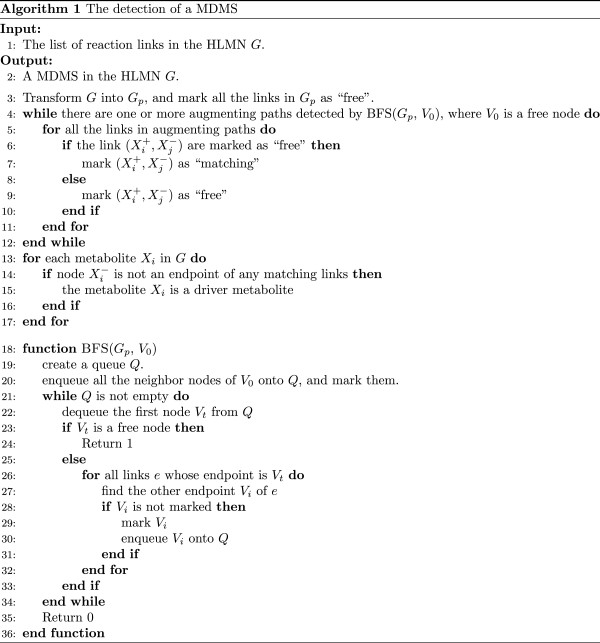
Pseudocode of the algorithm to determine a MDMS.

The HLMN is denoted by network *G* = (*X*,*R*), where *X* is the set of metabolite nodes, and *R* is the set of reaction links. The network *G* = (*X*,*R*) can be transformed into a bipartite network *G*_*p*_ = (*X*^+^,*X*^-^,*E*), where each node *X*_*i*_ is represented by two nodes $X_{i}^{+}$ and $X_{i}^{-}$, and each link *X*_*i*_ → *X*_*j*_ is represented as an undirected link $\left(X_{i}^{+},X_{j}^{-}\right)$[16,66]. Given a matching *M* in *G*_*p*_, the links in *M* are matching links, and the others are free. The node which is not an endpoint of any matching link is called free node. Simple paths are the path whose links are alternately matching and free. Augmenting path is a simple path whose endpoints are both free nodes. If there is a augmenting path *P*, *M* ⊗ *P* is a matching, where ⊗ is the symmetric difference operation of two sets. The size of the matching *M* ⊗ *P* is greater than the size of *M* by one. A matching is maximum if there are no augmenting paths. We used the well-known Hopcroft-Karp algorithm [67] to find maximum matchings in the bipartite network. For each maximum matching that we find, we can obtain a corresponding MDMS as illustrated in Figure 6. The pseudocode of the algorithm to detect a MDMS is shown in Figure 7. Different order of the link list could result in different initial matching set, which could further result in different maximum matching set. Thus, different MDMSs could be obtained. We compared every two of these MDMSs to make sure that the MDMSs are different from each other.

### Measures of centrality

Betweenness centrality quantifies the number of times a node acts as a bridge along the shortest path between two other nodes. Betweenness of node *v* is defined as

(1) $$B_{v}=\overset{}{\underset{s\neq v\neq t}{\sum }}[\sigma_{\text{st}}(v)/\sigma_{\text{st}}],$$

where *σ*_*st*_ is the number of shortest paths from node *s* to node *t*, and *σ*_*st*_(*v*) is the number of those paths that pass through *v*.

Out-closeness centrality of node *v* measures how fast it takes to spread information from *v* to other nodes. The out-closeness of node *v* is defined as

(2) $${\text{Cout}}_{v}=\overset{}{\underset{i\neq v}{\sum }}[1/d(v,i)],v\neq i,$$

where *d*(*v*,*i*) is the length of shortest path from node *v* to node *i*.

In-closeness centrality of node *v* measures how fast it takes to receive information from other nodes. The in-closeness of node *v* is defined as

(3) $${\text{Cin}}_{v}=\overset{}{\underset{i\neq v}{\sum }}[1/d(i,v)],v\neq i,$$

where *d*(*i*,*v*) is the length of shortest path from node *i* and node *v*.

Closeness centrality of node *v* measures how fast it takes to exchange information between *v* and other nodes. The closeness of node *v* is defined as

(4) $$C_{v}=\overset{}{\underset{i\neq v}{\sum }}[1/d_{\text{undire}}(v,i)],v\neq i,$$

where *d*_*undire*_(*v*,*i*) is the length of shortest path between node *v* and node *i*. Closeness centrality is defined in undirected networks. When we have to compute the closeness of node *v* in a directed network, the directed network is regarded as an undirected network.

### Identification of modules

We divided the HLMN into modules by using the SA algorithm [53,57]. Specifically, the implement tool "netcarto-w" [57] is used to detect modules by maximizing the modularity of the objective network. For a given decomposition of a network, the modularity *M* of this decomposition is defined as the gap between the fraction of links within modules and the expect fraction of links if the links are connected with no structure difference:

(5) $$M=\overset{N_{M}}{\underset{s=1}{\sum }}\left[l_{s}/L-{\left(d_{s}/2L\right)}^{2}\right],$$

where *N*_*M*_ is the number of modules, *L* is the number of links in the network, *l*_*s*_ is the number of links between nodes in the module *s*, and *d*_*s*_ is the sum of the degrees of the nodes in module *s*. By this definition, we can conclude that a good decomposition of a network must comprise many within-module links and as few as possible between-module links. However, if we just try to minimize the number of between-module links (equivalently, maximize the number of within-module links), the optimal partition consists of a single module and no between-module links. Equation (5) addresses this difficulty by imposing that *M* = 0 if nodes are placed at random into modules or if all nodes are in the same module [57].

Let *C* = -*M*, where *M* is the modularity as defined in equation (5). We used the SA algorithm to minimize the value of *C*. This is achieved by introducing a computational temperature *T*, which starts at a high value, and slowly decreasing *T*, each step of the SA algorithm attempts to replace the current solution by a random solution. When temperature *T* is high, the dependency between the previous and current solution is almost random, which could reduce the probability of being stuck at local optima. As temperature *T* goes to zero, the better solution is selected with an increasing probability. In this way, the SA algorithm gradually reaches a deep minima. Specifically, at each temperature *T*, we perform a number of random updates and accept them with probability:

(6) $$p=\left\{\begin{array}{ll} 1, & \text{if}\;C_{f}\leq C_{i},\\ e^{-(C_{f}-C_{i})/T}, & \text{if}\;C_{f}>C_{i}, \end{array}\right)$$

where *C*_*f*_ is the value of objective function after the update and *C*_*i*_ is the value before the update. At each temperature *T*, we take *n*_*i*_ = *fS*^2^ individual node movements from one module to another and *n*_*c*_ = *fS* collective movements which involve either merging two modules or splitting a splitting a module, where *S* is the number of metabolites in the network, and *f* is the iteration factor, which determines how many movements to perform at each temperature, we typically chose *f* = 1 as it was recommended in [57]. After the movements are evaluated at a certain *T*, the temperature *T* decreases to *T*^′^ = *cT*, with *c* = 0.965, where *c* is the cooling factor, which determines the number of iterations. When temperature *T* reaches to 0, the algorithm stops.

### Assignment of the roles of nodes

The roles of nodes are assigned based on two parameters: the within-degree and the partition coefficient. Nodes with similar roles are expected to have the similar within-degree and the similar partition coefficient. The within-degree *z*_*i*_ measures how well-connected node *i* is to other nodes in the same module, which is defined as

(7) $$z_{i}=\left(k_{i}-{\bar{k}}_{s_{i}}\right)/\sigma_{k_{s_{i}}},$$

where *k*_*i*_ is the number of links of metabolite *i* connecting to other metabolites in its module *s*_*i*_, ${\bar{k}}_{s_{i}}$ is the average of *k* over all metabolites in module *s*_*i*_, and $\sigma_{k_{s_{i}}}$ is the standard deviation of *k* in *s*_*i*_.

The partition coefficient *P*_*i*_ measures how well-distributes the links of node *i* are among different modules. The participation coefficient of a node is therefore close to 1 if its links are uniformly distributed among all the modules and 0 if all its links are within its own module. The partition coefficient *P*_*i*_ is defined as

(8) $$P_{i}=1-\overset{N_{M}}{\underset{s=1}{\sum }}{\left(k_{\text{is}}/k_{i}\right)}^{2},$$

where *k*_*is*_ is the number of links of node *i* to nodes in the module *s*, and *k*_*i*_ is the total degree of node *i*.

According to the within-module degree *z*, the nodes with *z* ≥ 2.5 are classified as hubs and nodes with *z* < 2.5 are classified as non-hubs. Both hub and non-hub nodes are then more finely characterized by using the values of the participation coefficient. Non-hub nodes are divided into four classes: 

(R1) nodes with all their links within their module (*P* ≤ 0.05);

(R2) nodes with some links to other modules (0.05 < *P* ≤ 0.62);

(R3) nodes with many links to other modules (0.62 < *P* ≤ 0.80);

(R4) nodes with links homogeneously distributed among all modules (*P* > 0.80).

The hub nodes are divided into three classes: 

(R5) nodes with the vast majority of links within their module (*P* ≤ 0.30);

(R6) nodes with many links to most of the other modules (0.30 < *P* ≤ 0.75);

(R7) nodes with links homogeneously distributed among all modules (*P* > 0.75).

The thresholds above for classifying the nodes into different roles according to their position in the modularized network are suggested by [57]. These thresholds are heuristically determined and validated by studying the nodes of different roles in real metabolic networks.

### Classification of links

The HLMN *G* = (*X*,*R*) can be transformed into a bipartite network *G*_*p*_ = (*X*^+^,*X*^-^,*E*), where *X* is the set of nodes, and *R* is the set of links, each node *X*_*i*_ in set *X* is split into two nodes $X_{i}^{+}$ and $X_{i}^{-}$ in set *X*^+^ and set *X*^-^, each link *X*_*i*_ → *X*_*j*_ in *R* is represented by an undirected link $\left(X_{i}^{+},X_{j}^{-}\right)$ in *E*. Given a maximum matching *M* in *G*_*p*_, the links in *M* are called matching links, and others are called free links. The links in a simple path or a simple circle are alternately matching and free. Each link in *G*_*p*_ belongs to a simple path or a simple circle.

All links in the HLMN have been classified into critical, ordinary or redundant according to their contribution to the robustness of controllability. The critical links appear in all the maximum matchings; the redundant links never appear in any maximum matching; and the ordinary links appear in some but not all maximum matchings.

Although the critical, ordinary and redundant links are defined based on all the maximum matchings, they can be determined based on their topological properties in the bipartite network with an arbitrary maximum matching. A proposition has been given in [68]: given the bipartite network *Gp* = (*X*^+^,*X*^-^,*E*), a link belongs to some of but not all maximum matchings (ordinary), iff, for an arbitrary maximum matching *M*, it belongs to either an even simple path which begins at a free node, or an even simple cycle. For the other links, the links which belong to *M* are critical and the links which do not belong to *M* are redundant. Based on this proposition, the critical, ordinary and redundant links could be correctively classified and avoid the enumeration of all the maximum matchings.

**Figure 8:**
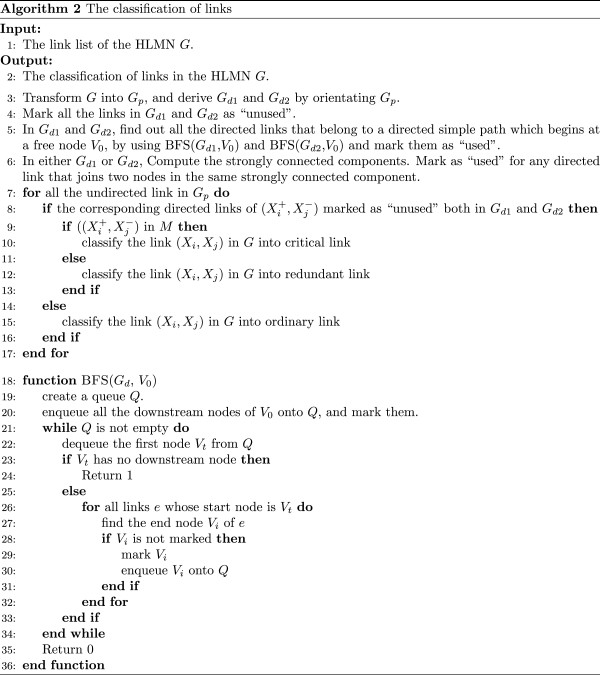
Pseudocode of the algorithm to classify links.

We used the link removing algorithm proposed by Régin [68] to classify the links in *G*. Given a maximum matching *M* in *G*_*p*_, we got two orientated bipartite networks *G*_*d*1_ = (*X*^+^,*X*^-^,*E*_*d*1_) and *G*_*d*2_ = (*X*^+^,*X*^-^,*E*_*d*2_), by orientating the bipartite network *G*_*p*_ = (*X*^+^,*X*^-^,*E*). *G*_*d*1_ was obtained by orientating the matching link $\left(X_{i}^{+},X_{j}^{-}\right)$ from $X_{i}^{+}$ to $X_{j}^{-}$, and the free link $\left(X_{k}^{+},X_{l}^{-}\right)$ from $X_{l}^{-}$ to $X_{k}^{+}$; *G*_*d*2_ was obtained in an opposite way of orientating links. We detected all simple paths which start from a free node in *G*_*d*1_ and *G*_*d*2_, and then computed the strongly connected components in either *G*_*d*1_ or *G*_*d*2_. The strongly connected components in *G*_*d*1_ or *G*_*d*2_ are simple circles because the links in maximum matching do not share same endpoints. If a link from *G*_*d*1_ or *G*_*d*2_ is in a simple path or a strongly connected component, then it is ordinary. For other links from *G*_*d*1_ or *G*_*d*2_: the link is critical if it is in the maximum matching *M*; if not, it is redundant. The pseudocode of the algorithm to classify links is shown in Figure 8.

### Chi-square test

The common test statistics include Z-tests, T-tests, Chi-squared tests and F-tests. Z-tests and T-tests are appropriate for comparing means under different conditions. F-tests are commonly used to decide whether groupings of data are meaningful by using analysis of variance. Chi-squared tests are commonly applied to sets of categorical data for various purpose, one of which is to establish whether or not an observed frequency distribution differs from a expected distribution. In this work, we do not care about the mean or the variance of a data set. We only care about wether the observed frequency distribution of one typical set is different from that in the whole network, which is the expected distribution. Thus, we chose chi-square test to test significance.

Chi-square test is used to determine whether there is a significant difference between the expected data and the observed data in one or more categories. The observed data is denoted by *O*_*i*_, where *i* = 1,2,…,*N*, and *N* is the number of categories. The expected data is denoted by *E*_*i*_, and $E_{i}=p_{i}\sum_{i=1}^{N}O_{i}$, where *p*_*i*_ is the expected percentage. The chi-square formula is defined as:

(9) $$\chi^{2}=\overset{N}{\underset{i=1}{\sum }}{\left(O_{i}-E_{i}\right)}^{2}/E_{i}.$$

We take the comparison between the percentages of different degree (low, medium and high) in the set A and those in the whole network, to illustrate the process of chi-square test. There are three categories of metabolites, which are the low, medium and high degree metabolites. The observed data are the number of the low, medium and high degree metabolites in the set A, which are 248, 188 and 137, respectively. The expected percentages are the percentages of the low, medium and high degree metabolites in the whole network, which are 33.3%, 33.4% and 33.3%, respectively. The chi-square statistic is computed using the above chi-square formula, as shown in Table 3. The chi-square statistic value here is 32.37.

**Table 3:** Worksheet for chi-square statistic computing

**Category**	***O***_***i***_	***p***_***i***_	***E***_***i***_	**(*O***_***i***_**-*E***_***i***_**)**^**2**^**/*E***_***i***_
*D*_*h*_	137	33.3%	190.81	15.17
*D*_*m*_	188	33.4%	191.38	0.06
*D*_*l*_	248	33.3%	190.81	17.14
Sum	573	1	573	32.37

After calculating the chi-square statistic value, we have to find the degrees of freedom. Degree of freedom refers to the number of percentage values that are free to vary, under the restriction that the sum of all the percentages are fixed. Obviously, the degree of freedom is *N* - 1, if the number of categories is *N*. There are three categories in this example, so the degree of freedom is two.

With the degree of freedom 2 and the predetermined level of significance 0.05, we can find the table value for chi-square statistic from the chi-square table ( http://www.unc.edu/~farkouh/usefull/chi.html) is 5.99. If the calculated chi-square value is equal to or greater than the table value, then the difference between the percentages among different sets is not due to chance alone. In this example, the calculated value of chi-square is 32.37, which is greater than the table value 5.99. It means that there is a significant difference between the fractions of the low, medium and high degree metabolites among the set A and those among the whole network.

## Abbreviations

HLMN: Human liver metabolic network; MDMS: Minimum driver metabolites set; UM: Universal metabolites; FEM: Functional essential metabolites; EUM: Essentiality undiscovered metabolites.

## Competing interests

The authors declare that they have no competing interests.

## Authors’ contributions

XL and LP conceived and designed the studies and wrote the manuscript. XL collected and analyzed the data and performed the experiments. Both authors read and approved the final manuscript.

## Supplementary Material

Additional file 1 **Table S1.** The list of metabolites and reactions in the human liver metabolic networks. Click here for file

Additional file 2 **Table S2.** 5000 maximum matchings and their corresponding minimum sets of driver metabolites. Click here for file

Additional file 3 **Table S3.** The frequencies of each metabolite in 51 different families of minimum driver metabolite sets. Click here for file

Additional file 4 **Additional notes and figures.** Property analysis for the driver metabolites determined based on the sampling method proposed by Jia et al. and connections between the control centrality and the human liver metabolism. Click here for file

Additional file 5 **Table S4.** The frequencies of each node acts as a driver node based on the sampling method proposed by Jia et al. Click here for file

Additional file 6 **Table S5.** The control centrality of each node in the human liver metabolic network. Click here for file
