# Supplementary material for: Detection of driver metabolites in the human liver metabolic network using structural controllability analysis
Source: BMC Syst Biol. 2014 May 3;8:51. doi: 10.1186/1752-0509-8-51 (PMC4024020; doi:10.1186/1752-0509-8-51)
Supplement: Additional file 4 — Additional notes and figures. Property analysis for the driver metabolites determined based on the sampling method proposed by Jia et al. and connections between the control centrality and the human liver metabolism. [file 1752-0509-8-51-S4.pdf]

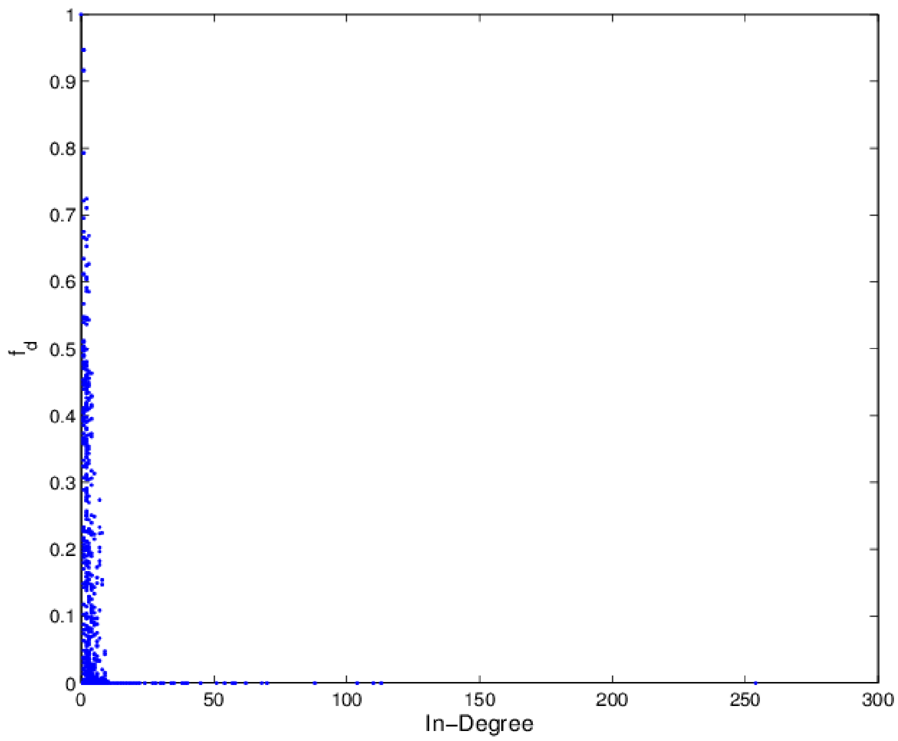


**Figure S1 Relationship between the frequency $f_d$ that each node acts a driver node and its in-degree**. The frequency $f_d$ decrease quickly with the in-degree in the human liver metabolic network.

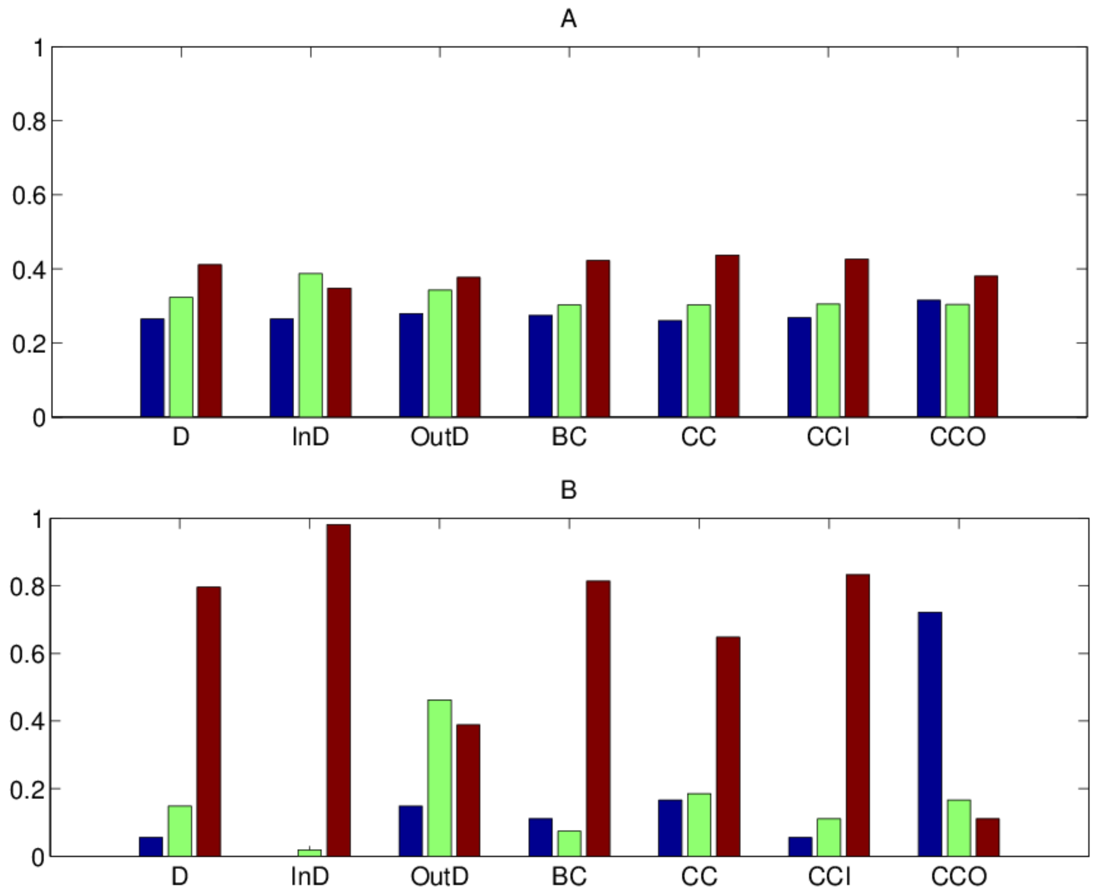


**Figure S2 Topological analysis of the driver metabolites which are detected based on the sampling method proposed by Jia et al..** A) The metabolites in the set A (metabolites whose frequency $f_d > 0$); B) The metabolites in the set B (metabolites whose frequency $1 > f_d \geq 0.6$). For the labels in the horizontal axis, ``D'', ``InD'', ``OutD'', ``BC'', ``CC'', ``CCI'', ``CCO'' respectively represent degree, in-degree, out-degree, betweenness, closeness, in-closeness, out-closeness. The hight of blue, green and brown bars respectively represent the fractions of the driver metabolites with high, medium and low centrality scores. The difference between the fractions for each centrality in B) is greater than that in A). Except for the bars of ``CCO'' in B), the brown bars are all lower than the blue and green ones for the same centrality , which indicates that the driver metabolites tend to avoid nodes with the high degree (resp., out-degree, in-degrees, betweenness, closeness and in-closeness), and the critical and high-frequency driver metabolites tend to have high out-closeness.

**Table S6 The chi-square statistic value for different centralities in set A and set B based on the sampling method proposed by Jia et al..**

| | D | InD | OutD | BC | CC | CCI | CCO |
|---|---|---|---|---|---|---|---|
| Set A | 20.08 | 14.11 | 9.02 | 22.97 | 31.76 | 25.19 | 6.38 |
| Set B | 52.87 | 102.27 | 8.74 | 56.55 | 24.17 | 61.11 | 37.08 |

The chi-square statistic values are all bigger than the table value 5.99.

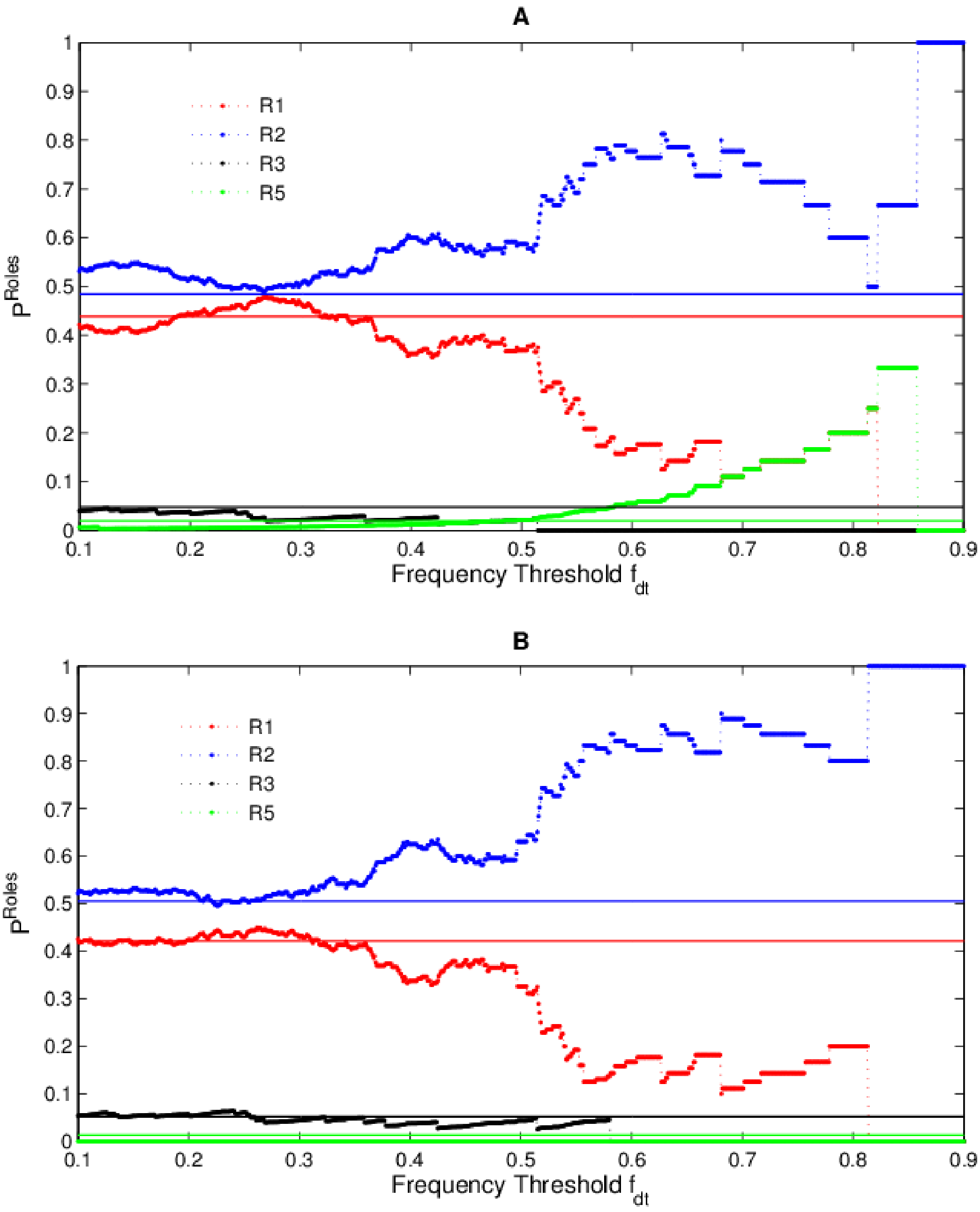


**Figure S3 The fractions of the metabolites with different roles based on different threshold.** The frequency $f_d$ of each node is determined based on the sampling method proposed by Jia et al.. A) and B) respectively show the results which are based on the modules detected by the SA algorithm and fast greedy algorithm. Each point connected by dotted lines is the fraction of the metabolites with a specific role among the set of driver metabolites whose frequency $1 > f_d \geq f_{dt}$, while each solid line means the fraction of metabolites with each role among the HLMN. With the frequency threshold $f_{dt}$ increasing, the fraction of R1 metabolites among the set of metabolites with $1 > f_d \geq f_{dt}$ decreases while the fraction of R2 metabolites increases, which shows the similar tendency with that based on the 5000 MDMSs.

**Additional Notes:**

The control centrality of a node measures the number of nodes that can be independently controlled by controlling this node alone. We attempted to reveal the possible connections between the control centrality and the actual importance of a metabolite in the HLMN, but we find there is no such connection, for the following explainations:

As shown in Table S5, the control centralities for the nodes in the human liver metabolic network are mainly distributed in two small regions (control centrality = 0.8441, 0.8449, 0.8456, 0.8463, 0.8471) and (control centrality = 0.0007, 0.0015, 0.0022). Moreover, more than 90% nodes' control centralities are among the region (control centrality =0.8441, 0.8449, 0.8456, 0.8463, 0.8471). For the nodes of relatively low control centralities (0.0007, 0.0015, 0.0022), we find that some metabolites are essential, for example alpha-tocopherol and gamma-tocopherol while some are not like carbon monoxide. It is difficult to connect the actual importance of metabolites to the relatively low control centralities. If there is any possible connections between the control centrality and the actual importance of metabolites, then more than 90% of the nodes in the human liver metabolic network are important, which is unrealistic and could not provide valuable informations on the human liver metabolic network.

Moreover, in this study, we mainly explored the possible connections between the frequency $f_d$ that a node acts as a driver node and the node's actual importance. Some connections have been revealed. We further investigate whether there is connections between the control centrality and the frequency $f_d$ in the human liver metabolic network. By comparing each node's control centrality and its frequency that a node acts as a driver node, we find that there is no significant correlation between these two metrics in the network, as shown in Figure S4.

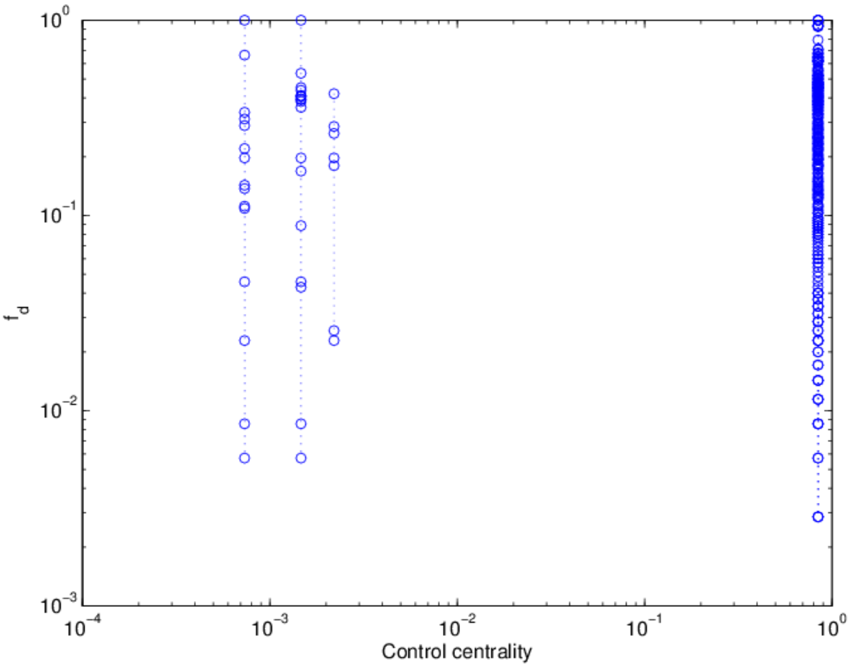


**Figure S4 Relationship between the frequency $f_d$ that each node acts a driver node and control centrality**.
